# Salvianolic acid a inhibits platelet activation and aggregation in patients with type 2 diabetes mellitus

**DOI:** 10.1186/s12872-019-01316-z

**Published:** 2020-01-13

**Authors:** Ai-ming Zhou, Yi-jia Xiang, En-qian Liu, Chang-hong Cai, Yong-hui Wu, Le-bing Yang, Chun-lai Zeng

**Affiliations:** 1 The First Affiliated Hospital of Wenzhou Medical University, Wenzhou, 325000 Zhejiang China; 2 Department of Cardiology, Lishui Hospital, Zhejiang University School of Medicine, Lishui, 323000 Zhejiang China; 3 Zhejiang University School of Medicine, Hangzhou, 310029 Zhejiang China; 4 The Fifth Affiliated Hospital of Wenzhou Medical University, Lishui, 323000 Zhejiang China

**Keywords:** Type 2 diabetes mellitus, Salvianolic acid a, PAC-1, CD62p, Maximal platelet aggregation

## Abstract

**Background:**

Platelets in patients with type 2 diabetes mellitus (DM2) are characterized by increased activation and aggregation, which tends to be associated with a high morbidity and mortality due to cardiovascular disease (CVD). Moreover, a large proportion of DM2 patients show an inadequate response to standard antiplatelet treatments, contributing to recurrent cardiovascular events. In our previous study, we indicated that Salvianolic acid A (SAA) presents an antiplatelet effect in healthy volunteers. However, whether it can inhibit “activated platelets” with a pathologic status has not been explored. Therefore, this study was designed to investigate the antiplatelet effect of SAA and its diabetic complication-related difference in DM2.

**Methods:**

Forty patients diagnosed with DM2 from January 2018 to April 2018 were recruited. Fibrinogen-binding (PAC-1) and P-selectin (CD62p) flow cytometry reagents were measured under resting and stimulated conditions by flow cytometry, while agonist-induced platelet aggregation was conducted by light transmission aggregometry. Before all these measurements were conducted, all platelet samples were preincubated with a vehicle or SAA for 10 min. Additionally, the diabetic complication-related difference in the antiplatelet effect of SAA was further studied in enrolled patients.

**Results:**

The expressions of PAC-1 and CD62p were elevated in DM2, as well as the maximal platelet aggregation. In addition, SAA decreased the expressions of PAC-1 and CD62p, which were enhanced by ADP and thrombin (all *P* < 0.01). It also reduced the platelet aggregation induced by ADP (*P* < 0.001) and thrombin (*P* < 0.05). Comparing the antiplatelet effect of SAA on DM2, with and without diabetic complications, no statistically significant difference was found (all *P* > 0.05).

**Conclusions:**

The present study demonstrated that SAA can inhibit platelet activation and aggregation in patients with DM2, and the inhibition did not abate for the existence of diabetic complications.

## Background

Diabetes mellitus (DM), defined by elevated glycemic markers, has reached an epidemic level worldwide, and its prevalence continues to climb. According to the latest IDF Diabetes Atlas report, there was an estimated 425 million cases of diabetes among adults aged 20–79 years in 2017, and this figure is expected to increase to 629 million by 2045 [1]. Besides, patients suffering from DM have a substantially increased risk of cardiovascular events, compared to individuals without diabetes [2]. Notably, cardiovascular disease (CVD) is a significant cause of morbidity and mortality in this population, which has led to diabetes being called a “cardiovascular disease equivalent” [3].

It is well known that platelet hyperactivity plays a pivotal role in the initiation of chronic medical conditions, such as atherosclerosis, coronary vascular disease and cerebrovascular disease. Moreover, the platelets of subjects with DM are in a state of high platelet reactivity and activation [4]. Currently, there are three main antiplatelet drugs for different platelet signaling pathways: cyclooxygenase-1 (COX-1) inhibitors, GP IIb/IIIa inhibitors and the adenosine diphosphate (ADP) P2Y12 receptor antagonist [5]. However, a large proportion of DM patients, presenting “aspirin resistance” [6, 7] and the up-regulation of P2Y12 receptors, have a poor responsiveness to current antiplatelet agents, resulting in a high rate of adverse recurrent cardiovascular events. Therefore, new antiplatelet agents are needed in to reduce the CVD risk of DM patients.

Salvianolic acid A (SAA) is a minor phenolic carboxylic acid, extracted from Danshen, which exhibits a variety of pharmacological activities, such as anti-oxidative [8], cardio-protective [9], neuroprotective [10], antidiabetic [11], anti-inflammatory [12], and anti-fibrotic effects [13]. Our previous study showed that SAA can inhibit platelet activation and aggregation in healthy volunteers [14]. Nevertheless, whether SAA can inhibit ‘activated platelets’ has not been extensively researched. Therefore, the aim of this study was to investigate the antiplatelet effect of SAA and its complication-related difference in patients with DM2.

## Materials and methods

### Participants

All consecutive patients diagnosed with DM2 in the Department of Endocrinology of the Central Hospital of Lishui from January 2018 to April 2018 were recruited in this study. The inclusion criteria were as follows: (a) subjects older than 18 years and willing to provide informed consent; (b) subjects with a known diagnosis of diabetes; and (c) subjects without a history of taking any antiplatelet or anticoagulant agents during the 2 weeks preceding the venipuncture. The exclusion criteria were as follows: (a) hemodynamically unstable subjects; (b) subjects with documented life threatening diseases (malignancy, HIV/AIDS, etc.); (c) subjects on maintenance hemodialysis; (d) subjects with a platelet count outside of the normal range; and (e) subjects without complete clinical data (fundus examination, electroneuromyography, glomerular filtration rate, etc.).

Four milliliters of blood were drawn from each participant into the siliconized vacutainer, containing 1:9 (v/v) 3.8% sodium citrate, in the morning (between 6 am and 8 am). Then, the expressions of the platelet activation markers and the maximal platelet aggregation were measured by flow cytometry and light transmission aggregometry, respectively.

After hospitalization, a fundus examination, electroneuromyography and a glomerular filtration rate test were performed on all participants to clarify the diagnosis of diabetic complications. According to the diabetic complications, the participants were divided into three groups: DM2 without complications (DM2 group), DM2 with diabetic retinopathy (DRP) or diabetic peripheral neuropathy (DPN) (DRP/DPN group), and DM2 with diabetic nephropathy (DN) (DN group). Then, complication-related differences among these three groups were further studied. Diabetic complications were diagnosed according to the latest guidelines concerning diabetic complications. The protocol and all procedures were approved by the Ethics Committee of the Central Hospital of Lishui City. Written informed consent was obtained from all of the participants.

### Materials

SAA was purchased from Plant Bio-Engineering Co. Ltd. (Purity 98%, Xi’an, China). Flow cytometry reagents, such as anti-CD62p/PE and PAC-1/FITC, were obtained from Becton Dickinson (San Jose, CA, USA). ADP and thrombin were purchased from Helena Laboratories (Beaumont, TX, USA) and Xinfan Bio-technology Co, Ltd. (Hangzhou, China), respectively. All other chemicals were of analytical grade or products of the highest quality available.

### Platelet aggregation

Agonist-induced maximum platelet aggregation was measured using light transmittance aggregometry (AggRAM, Helena Laboratories Inc., Beaumont, TX, USA). Citrate-anticoagulated whole blood was centrifuged at 1000 r for 10 min at room temperature to obtain platelet rich plasma (PRP). Platelet poor plasma (PPP) was obtained from the remaining specimen by re-centrifugation at 4000 r for 5 min. Then, PRP was preincubated with a vehicle or SAA (final concentration: 0.1 mg/ml) for 10 min at 37 °C, without stirring. Aggregation was performed using ADP (4 μM) or thrombin (0.4 U/ml), and the optical density was recorded for 5 min, as platelets began to aggregate. Aggregation is expressed as the percentage of maximal light transmittance, compared to the baseline, where the maximal is defined by the light transmittance of platelet-poor plasma.

### Flow cytometry

The surface marker expression on platelets in PRP was measured by a Beckman Coulter EPICS XL flow cytometer (Beckman Coulter, Hialeah, FL, USA). The PRP sample (5 μl) was added to 45 μl of PBS, containing appropriately diluted fluorescent MAbs for the detection of the platelet PAC-1 expression and CD62p expression. All the samples were preincubated with a vehicle or SAA (0.1 mg/ml) for 10 min at room temperature. A panel of agonists [ADP (20 μM) or thrombin (0.2 U/ml)] were then added into the SAA group and stimulation group. The samples were incubated at room temperature in the dark for 15 min and then diluted and mildly fixed with 0.1% (v/v) formaldehyde saline, before being analyzed. The platelet PAC-1 expression and CD62p expression were reported as the percentages of P-selectin-positive and fibrinogen binding-positive cells, respectively, in the platelet population.

### Statistical analysis

Continuous variables are presented as the means ± standard deviation and categorical variables, as percentages. The Shapiro-Wilk test was used to verify whether the data were normally distributed. The significance of the differences between the ADP group and SAA + ADP group, and between the Thrombin group and SAA + Thrombin group, was determined using the paired-sample T test or Wilcoxon’s signed rank test. Statistical significance was accepted at *P* < 0.05, and SPSS ver. 23 was used.

## Results

### Study population and clinical characteristics

Forty subjects (21 males and 19 females, aged 57.98 ± 8.19 years), who met the inclusion criteria and not the exclusion criteria, were enrolled in the study. Among them, 18 subjects had hypertension, and 27 subjects had hyperlipemia. According to the index of hemoglobin A1c (HbA1c), most of the participants enrolled in this study had a poor metabolic control in the last two or 3 months, before the study. Besides, the majority of the participants were receiving insulin therapy under a glycemic control. Details of the clinical characteristics of the individuals are shown in Table 1.

**Table 1:**
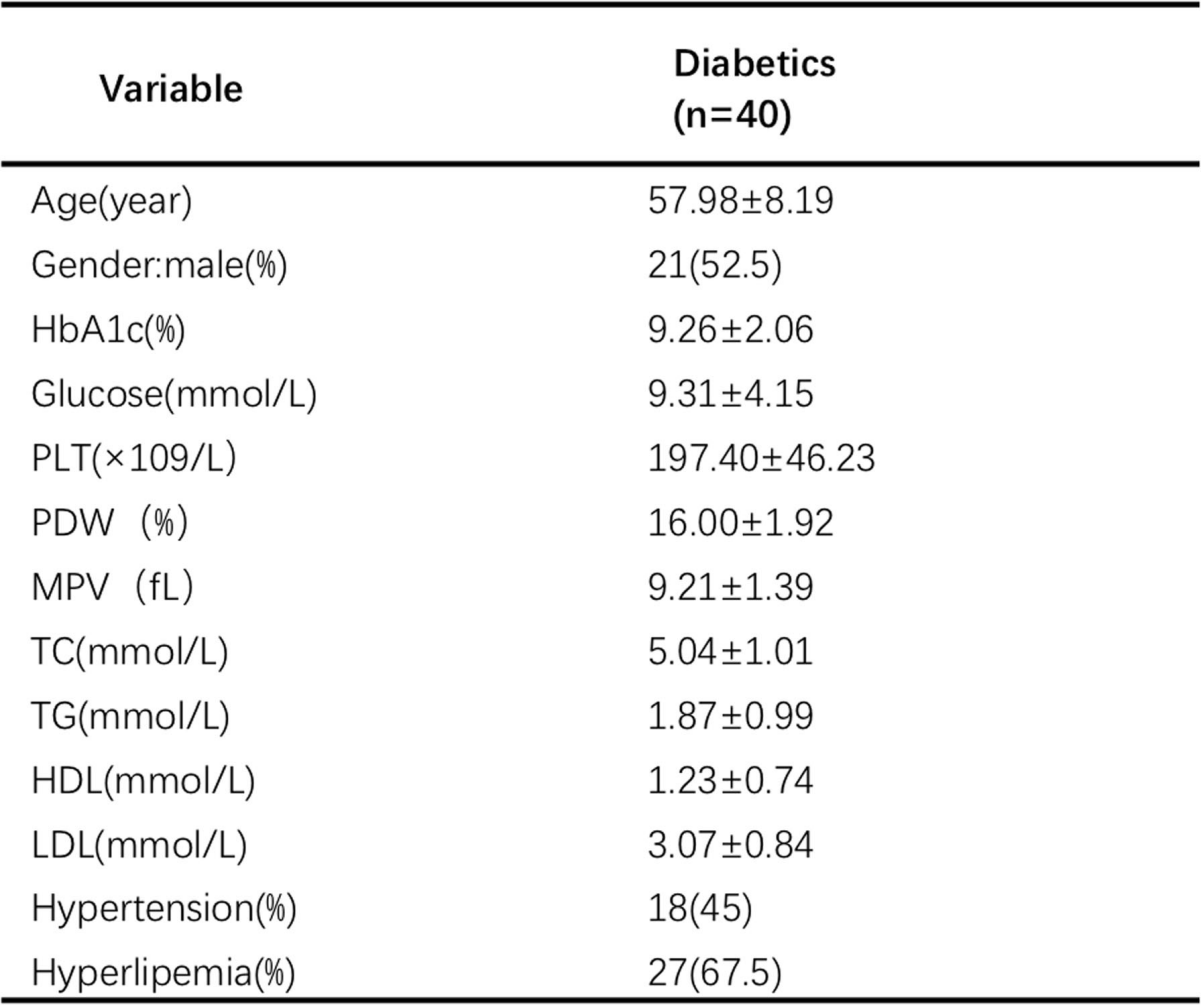
Anthropometric and biochemical characteristics of the subjects included in the study

The results are presented as the mean ± SD or n (%). *PLT* platelet; *PDW* platelet distribution width; *MPV* mean platelet volume; *TC* total cholesterol; *TG* triglyceride; *HDL* high-density lipoprotein; *LDL* low-density lipoprotein; *HbA1c* hemoglobin A1c

### Effects of SAA on platelet aggregation

**Fig. 1:**
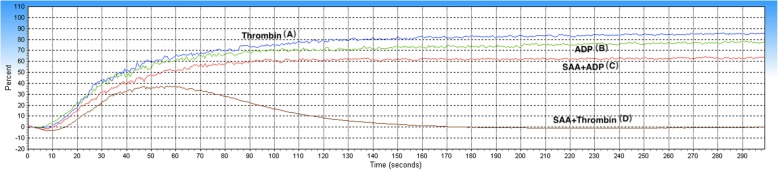
Representative curve graph of the maximal platelet aggregation. The maximal platelet aggregation was induced by ADP (4 μM) (B, C) or thrombin (0.4 U/ml) (A, D) in PRP, preincubated with a vehicle (A, B) or SAA (C, D) (0.1 mg/ml; 22°C; 10 min)

**Fig. 2:**
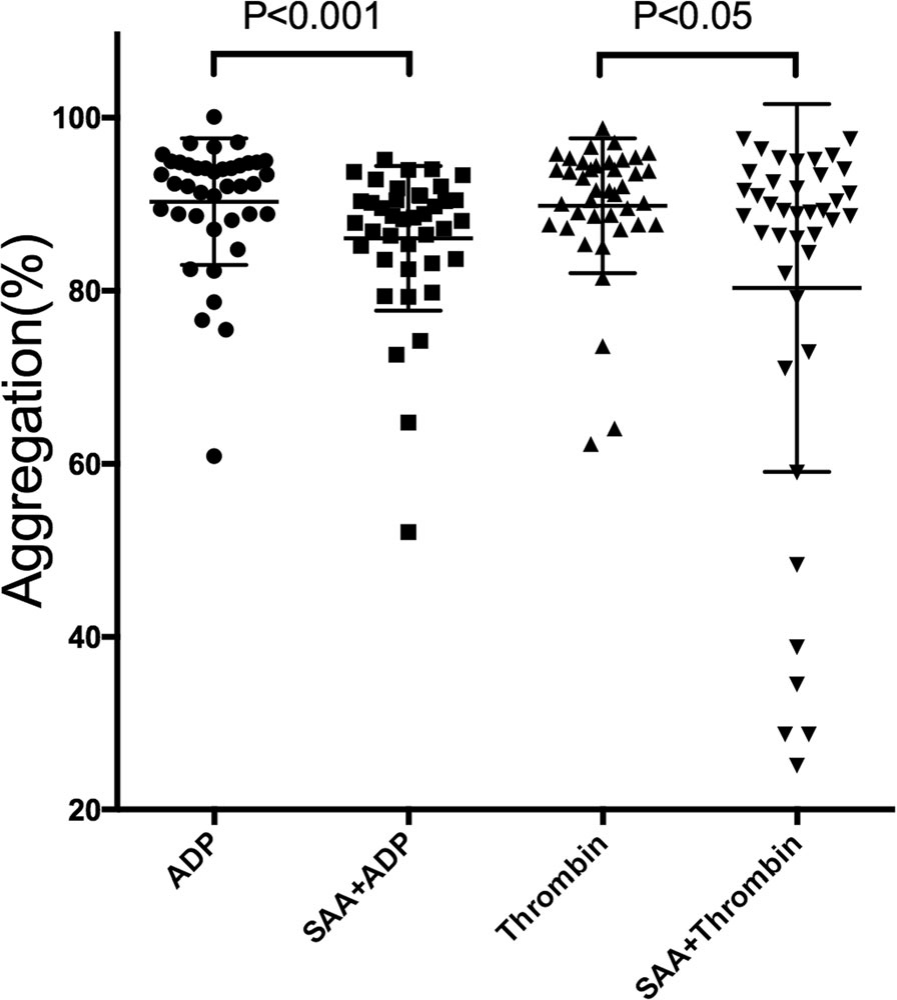
Effects of Salvianolic acid A (SAA) on platelet aggregation. Platelet-rich plasma (PRP) was preincubated for 10 min with SAA (0.1 mg/ml) or a vehicle. Platelet aggregation was initiated with ADP (4 μM) or thrombin (0.4 U/ml)

The results showed that the mean maximal platelet aggregation was 90.33 ± 7.34% in the ADP group, 86.10 ± 8.38% in the SAA + ADP group, 89.10 ± 7.80% in the Thrombin group, and 80.34 ± 21.27% in the SAA + Thrombin group. The ADP- and Thrombin-induced maximal platelet aggregation was found to be significantly decreased in the SAA group (SAA + ADP group and SAA + Thrombin group), compared to the control groups (ADP group and Thrombin group). In other words, the platelet aggregation induced by ADP and thrombin was inhibited by SAA (Figs. 1, and 2).

### Impacts of SAA on agonist-induced single platelet activation

**Fig. 3:**
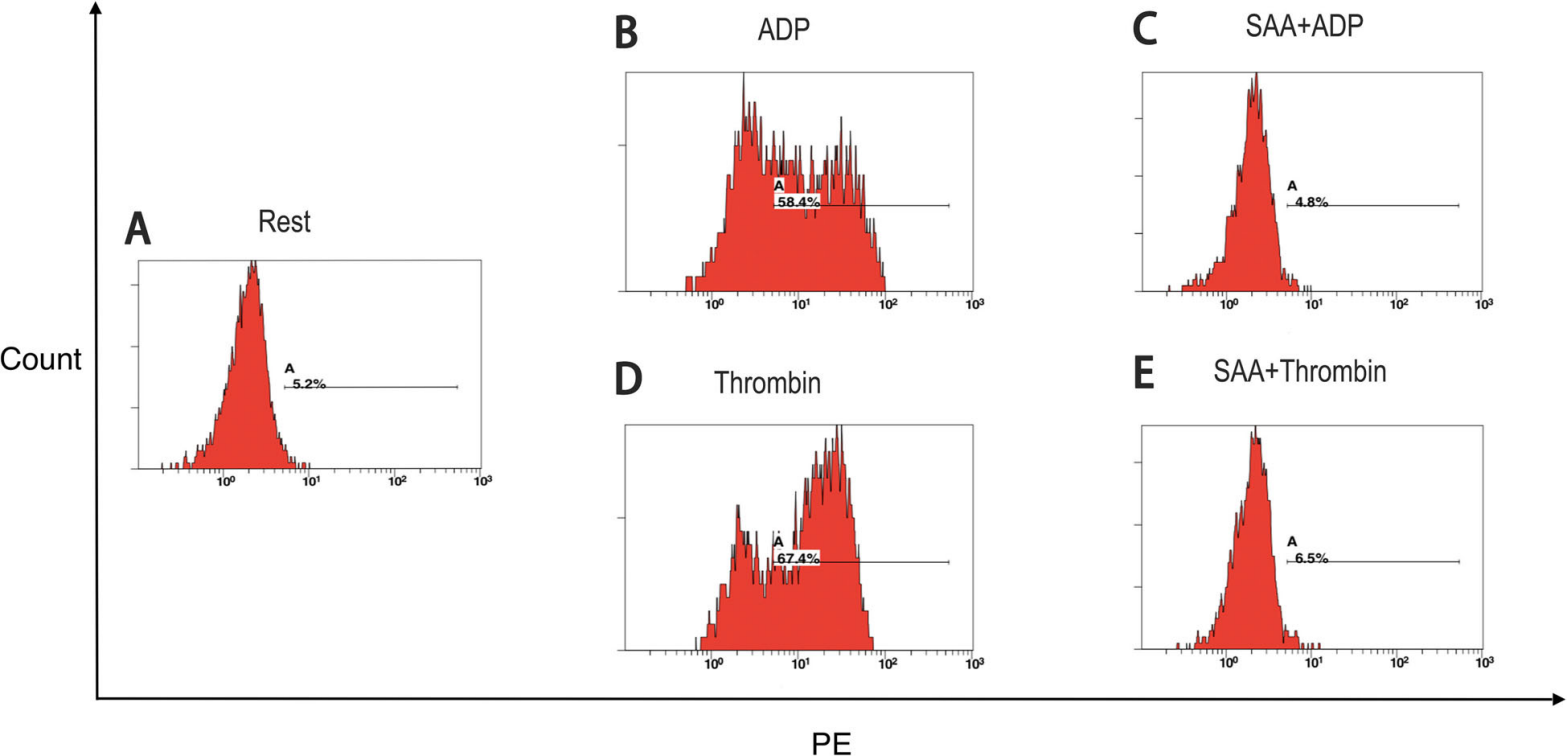
Representative histograms of the expression level of the active form of CD62p on non-stimulated platelets (shown as Rest) (**a**) and on platelets stimulated with ADP (**b**, **c**) or thrombin (**d**, **e**) in PRP, preincubated with a vehicle (**b**, **d**) or SAA (C, E) (0.1 mg/ml; 22 °C; 10 min). For each sample, 5000 platelets were acquired. The platelets were labeled with the monoclonal antibody, anti-CD62p (PE)

**Fig. 4:**
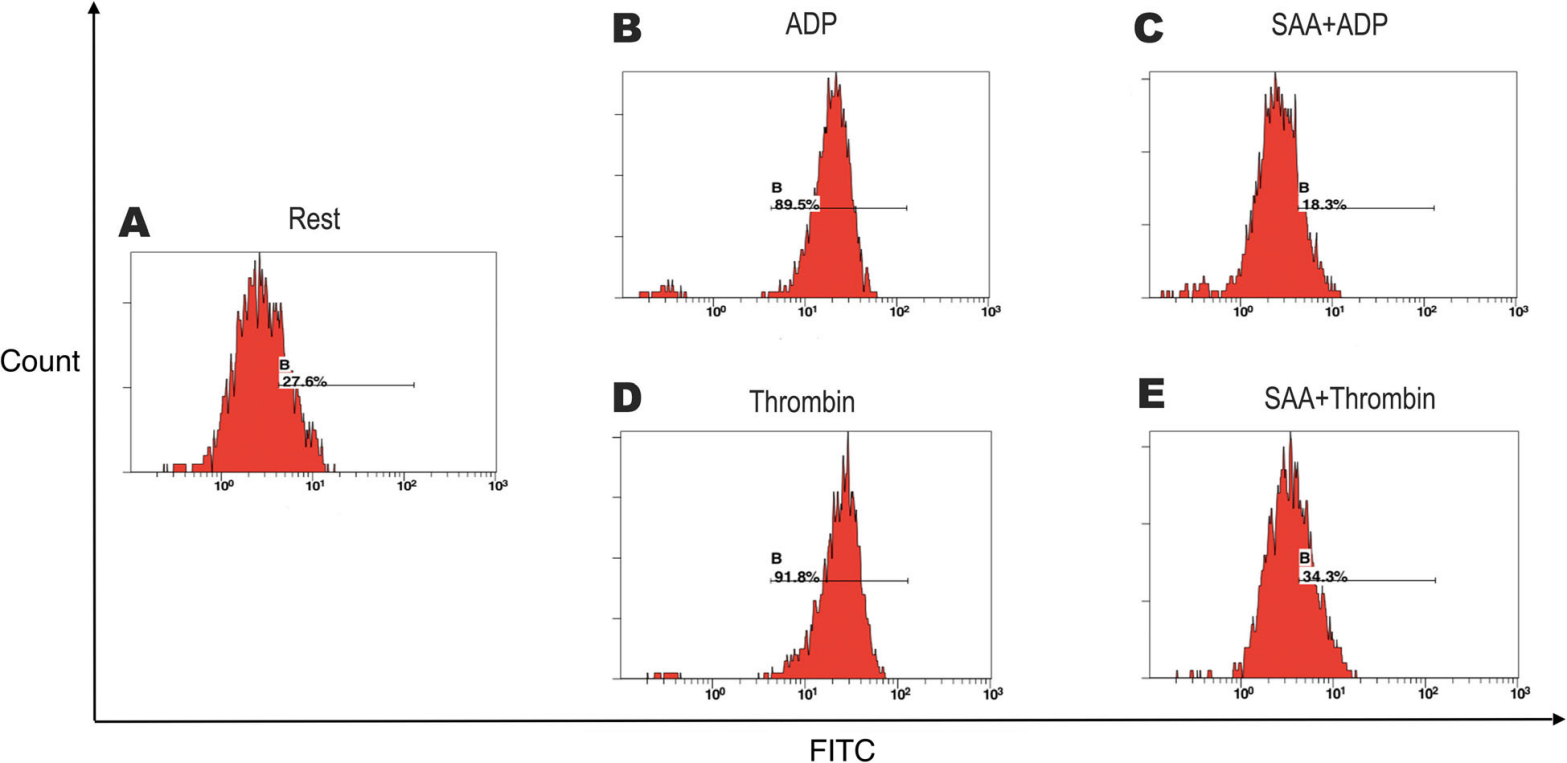
Representative histograms of the expression level of the active form of PAC-1 on non-stimulated platelets (shown as Rest) (**a**) and on platelets stimulated with ADP (**b**, **c**) or thrombin (**d**, **e**) in PRP, preincubated with a vehicle (**b**, **d**) or SAA (**c**, **e**) (0.1 mg/ml; 22 °C; 10 min). For each sample, 5000 platelets were acquired. The platelets were labeled with the monoclonal antibody, anti-PAC-1 (FITC)

**Fig. 5:**
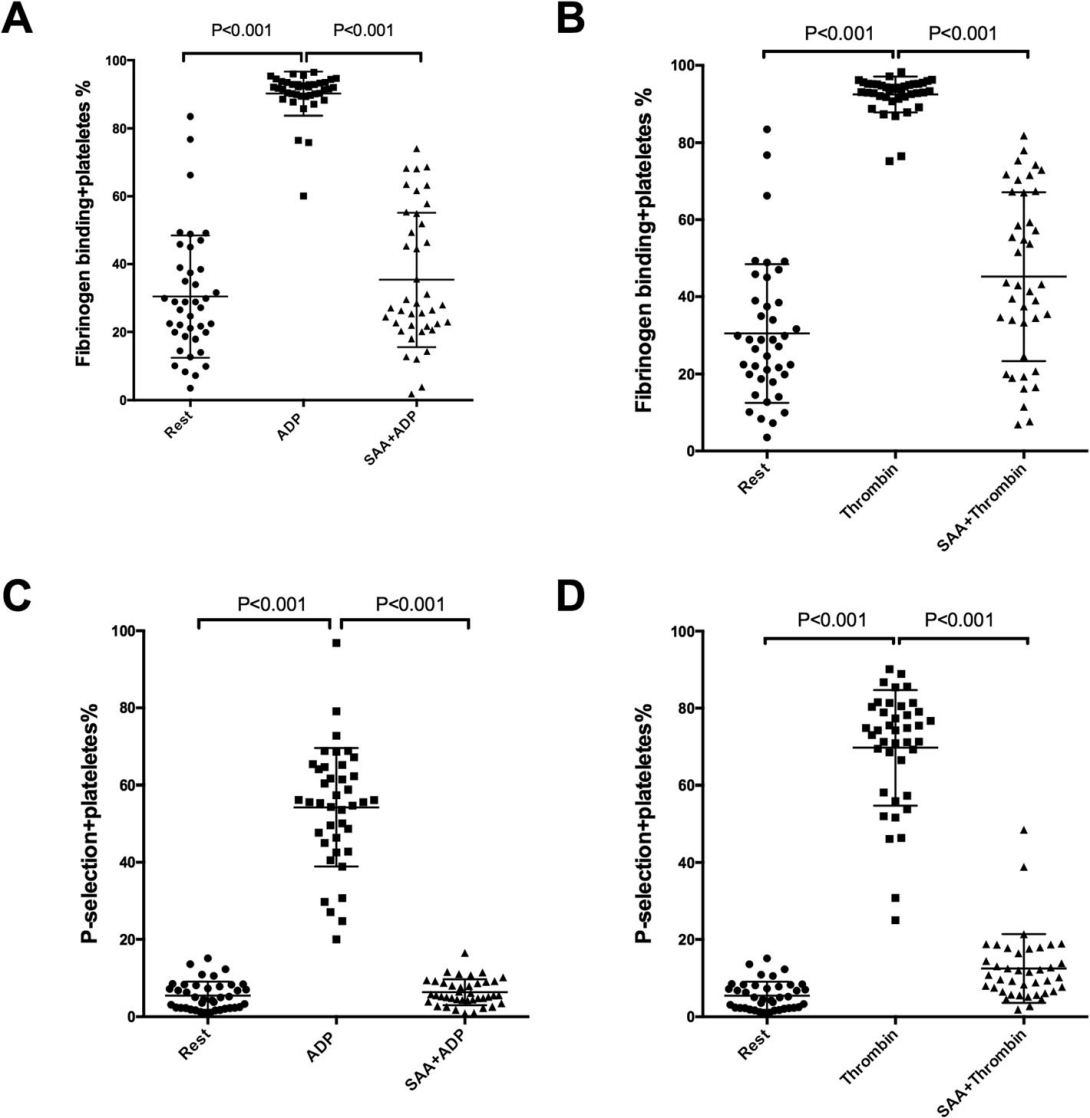
Impacts of Salvianolic acid A (SAA) on single-platelet activation**.** Platelet-rich plasma (PRP) was preincubated with a vehicle or SAA (0.1 mg/ml, 22 °C, 10 min) in the presence of the fluorescent PAC-1 or CD62p antibodies. Samples were then stimulated by ADP (20 μM) or thrombin (0.2 U/ml) and incubated for 15 min. The PAC-1 (**a**, **b**) and CD62p (**c**, **d**) of single platelets were monitored by flow cytometry

Further study was performed to explore the effects of SAA on single platelet activation using flow cytometry on PRP. ADP (20 μM) and thrombin (0.2 U/ml) increased PAC-1, a marker reflecting platelet aggregability, from 30.47 ± 17.97% at the baseline to 90.18 ± 6.46% and to 92.45 ± 4.65%, respectively (all *P* < 0.001). These increases were reduced to 35.41 ± 19.45% and 44.16 ± 23.29% in the presence of 0.1 mg/ml of SAA. Similar inhibitory effects of SAA were found on the ADP- and thrombin-induced platelet CD62p expression, which reflects platelet secretion (Figs. 3, 4, and 5).

### The diabetic complication-related difference in the antiplatelet effects of SAA

**Fig. 6:**
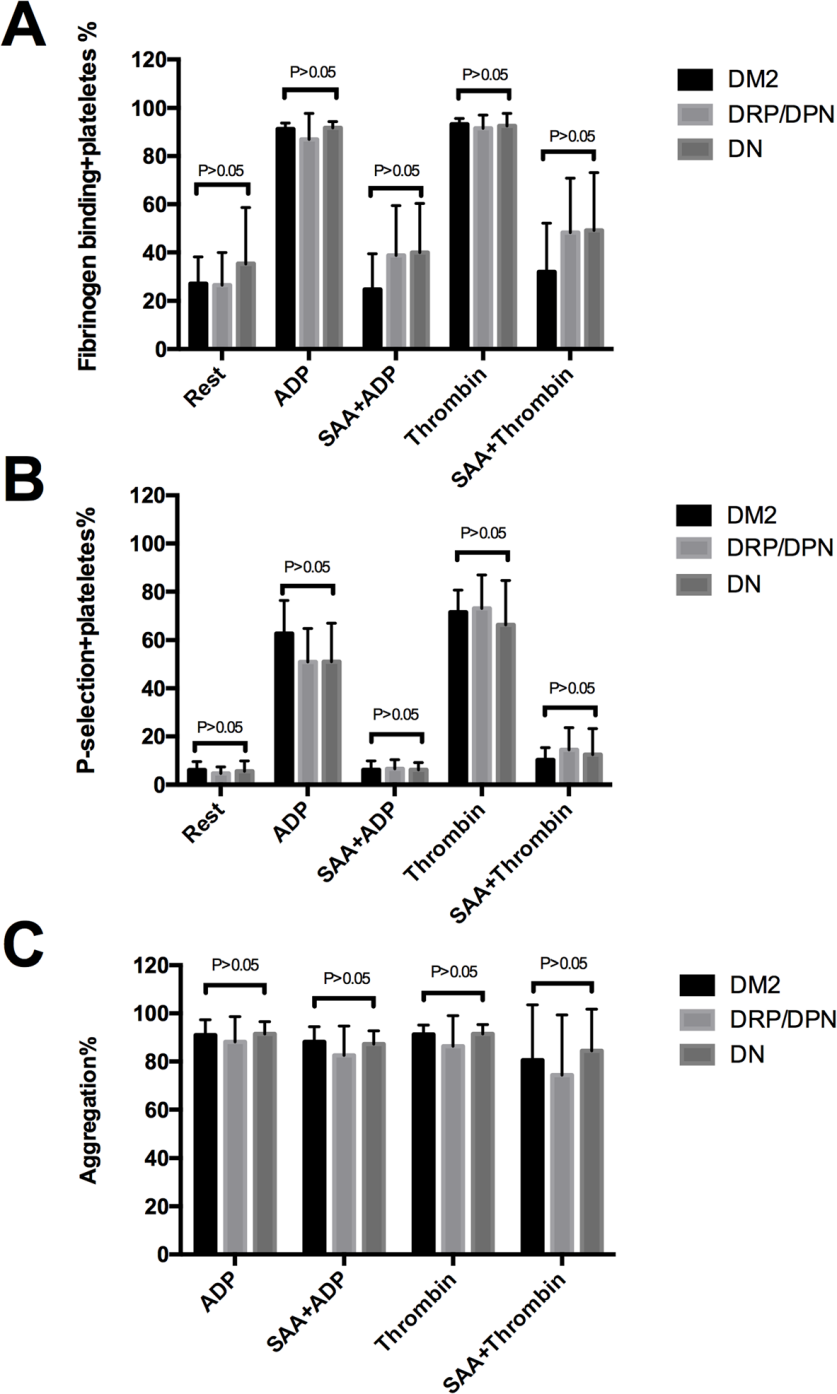
Antiplatelet effect of SAA on DM2, with or without diabetic complications. The expressions of PAC-1 (**a**) and CD62p (**b**) in the DM2 group, DRP/DPN group and DN group were not significantly different (*P* > 0.05). The effect of SAA on platelet aggregation (**c**) also showed no remarkable difference among these three groups (*P* > 0.05). DM2 group: DM2 without diabetic complications; DRP/DPN group: DM2 with diabetic retinopathy or diabetic peripheral neuropathy; DN group: DM2 with diabetic nephropathy

Among the 40 patients included in this study, 17 were diagnosed with DN (DN group), 12 with DRP or DPN (DRP/DPN group), whereas the remaining 11 participants did not have diabetic complications (DM2 group). There was no statistical difference among these three groups with regard to age, gender, total cholesterol (TC), triglycerides (TG), high-density lipoproteins (HDL), low-density lipoproteins (LDL), hemoglobin A1c (HbA1c), and fasting glucose level. The hypertension rate in the DN group was higher than that in the DM2 group (*P* < 0.05). The details of the clinical and biochemical baseline characteristics of the population studied are summarized in Table 2. For the effects of SAA on platelet aggregation, our study revealed that there were no remarkable differences among the DM2 group, DRP/DPN group and DN group (*P* > 0.05). Additionally, a comparison of the impacts of SAA on agonist-induced single platelet activation also showed no significant difference (all *P* > 0.05) (Fig. 6).

**Table 2:**
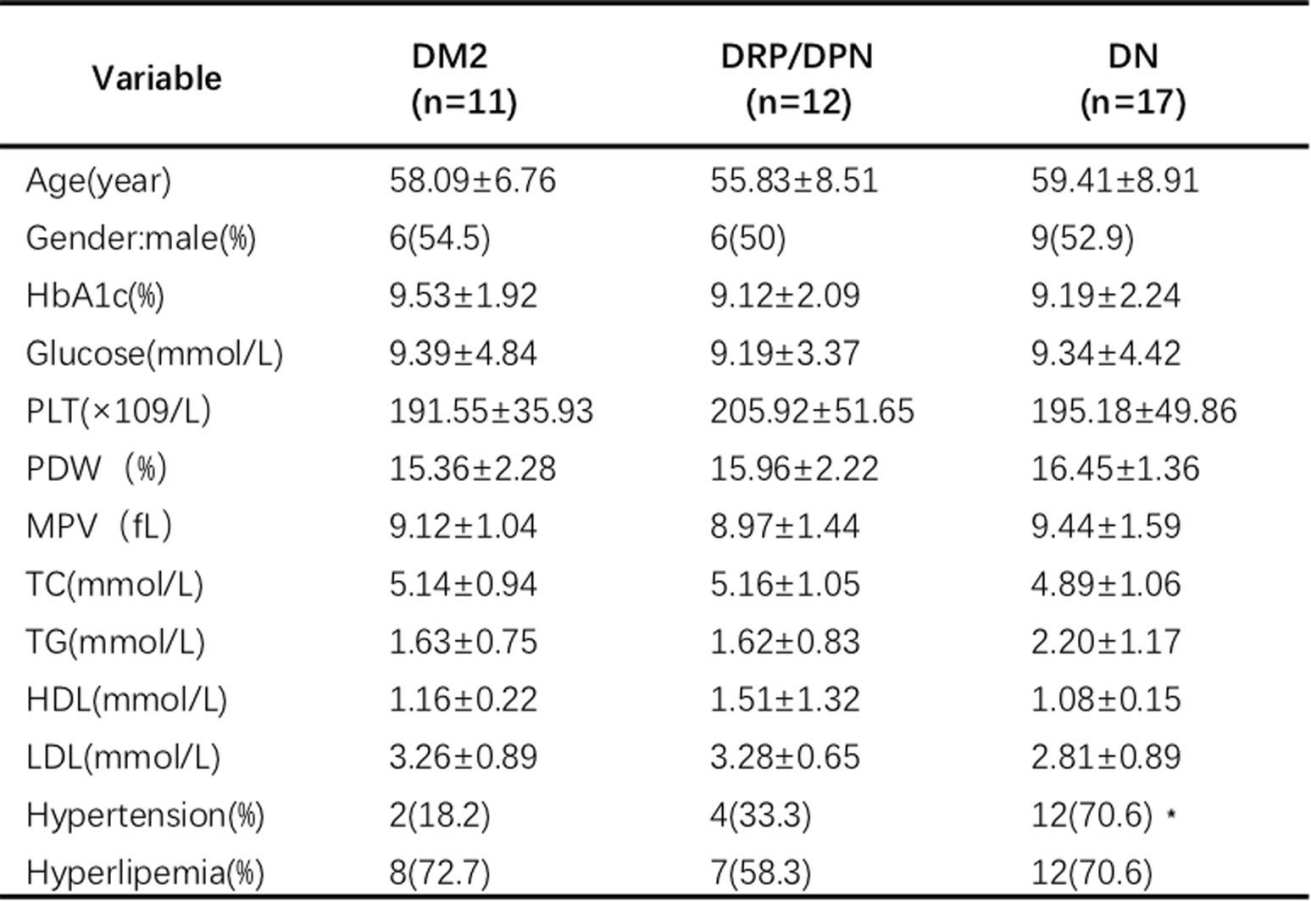
Baseline demographic data and clinical characteristics of the three groups included in the study

The results are presented as the mean ± SD or n (%). * *P* < 0.05, the DM2 group vs DM2 with the DN group. *PLT* platelet; *PDW* platelet distribution width; *MPV* mean platelet volume; *TC* total cholesterol; *TG* triglyceride; *HDL* high-density lipoprotein; *LDL* low-density lipoprotein; *HbA1c* hemoglobin A1c

## Discussion

In the present study, we demonstrated that SAA exhibits an antiplatelet effect on patients with DM2. Notably, this antiplatelet effect is not less effective in DM2 patients with complications than in those without complications. All these findings enable a better understanding of the antiplatelet effect of SAA in DM2, which ultimately leads to the development of novel pharmaceutical strategies for the antiplatelet treatment of patients suffering from DM2.

Platelets obtained from DM2 are hyperactive and demonstrate exaggerated aggregation as well as thrombus generation [15]. There are many different mechanisms that have been attributed to the diabetes-associated enhanced platelet activation [16], such as a loss of the antiplatelet effect of insulin [17], high blood glucose, oxidative stress [18], elevated vascular shear forces [19], increased binding of fibrinogen [20], altered expression of glycoprotein receptors and proteins attached to the platelet surface [21–23]. It is clear, from the literature, that there is an increased expression of platelet activation makers, such as CD62p and PAC-1, as measured by flow cytometry in DM2, which contributes to the progression of thrombotic and CVD events. Our study suggested that the expressions of CD62p and PAC-1 were elevated in DM2 under a resting condition, while the expressions of these two markers were evidently decreased, after preincubation with SAA in vitro, under a stimulating condition, compared to the control groups. Similar results were achieved in our previous study, which reported that platelet activation markers were at a low expression level in the SAA group in healthy volunteers [14]. It is worth noting, in our present study, that the expressions of PAC-1 and CD62p in DM2 under the resting condition were not increased to as high a level as they did in the previous study [24]. This might be associated with the dissimilarity of the participants. Most of the participants enrolled in this study were receiving insulin therapy under a glycemic control. Moreover, previous researches indicated that insulin treatment has a beneficial effect on platelet activation, and aggregation has a beneficial effect on “diabetic platelets”, which may be related to the direct action of insulin on erythrocyte deformability [25, 26]. This may explain why the level of platelet activation in our study is lower than that in other studies. Additionally, the present study revealed that the maximum platelet aggregation induced by ADP or Thrombin in the SAA group was also obviously inhibited. However, the inhibiting effect in this study was not as good as that in our previous study [14]. In order to fully embody the functional status of diabetic platelets in plasma, this study chose PRP as the test specimen, instead of washed platelets. However, there are more small-molecule plasma proteins, which may interact with SAA and ultimately interfere with the antiplatelet effect of SAA in PRP, rather than washed platelets, which may account for the different result. Based on this point, the interaction between plasma proteins and SAA should be considered, and the use of SAA therapy in DM2 patients should be promoted in the future.

Recent researches on the action mechanisms of SAA have found that SAA can antagonize the activity of both P2Y1 and P2Y12 receptors in the low μM range [27]. Moreover, pretreatment with SAA on platelets caused an increase in the cAMP level in platelets activated by ADP, which indicates that SAA might possess antithrombotic activities [28]. In our study, we found that SAA inhibited platelet activation aroused by a variety of agonists, which indicates that SAA may intervene in a shared signaling molecule of platelet activation. However, a limited number of studies have investigated the interruption of signaling events by SAA. According to our previous study, SAA inhibits platelet activation and arterial thrombosis via the inhibition of phosphoinositide 3-kinase (PI3K) [14]. Moreover, we found that SAA demonstrated a more potent inhibition of Rap1b activation than PI3KP inhibitors. As Rap1b is dually controlled by the PLC and PI3K pathways, SAA may affect other platelet signaling mechanisms, apart from PI3K pathways. Recent research has focused on the platelet-specific collagen receptor, glycoprotein VI (GPVI), as a potential antiplatelet target. Signaling events downstream from GPVI are influenced by hyperglycemia, oxidative stress, and shear stress [19]. According to previous investigations, SAA has extensive pharmacological effects, including antidiabetic [11], antioxidant [8] and other effects. Therefore, the antiplatelet effect of SAA may be associated with the interruption of the GPVI signaling pathway. To confirm this hypothesis, further research will be needed to assess this possible signaling pathway.

In addition, we further investigated the diabetic complication-related difference in platelet aggregation and activation in response to SAA. Our findings indicate that the antiplatelet efficacy of SAA was not reduced in DM2 with diabetic complications, comparing to DM2 without complications. In other words, the antiplatelet effect of SAA did not abate diabetic complications. Besides, the baseline of the platelet aggregation and activation between DM2 with and DM2 without complications shows no statistical difference, which may explain their similar response to SAA.

Since SAA possesses a variety of bioactivities, including a defense from oxidative damage, improvement of remembrance [28], lowering of blood glucose and inhibition of platelet aggregation and activation, patients with diabetic complications treated with SAA could not only gain cardiovascular benefits, but also additional benefits relating to diabetic complications control. On the other hand, SAA could also play a preventive role, when SAA is treated as an antiplatelet agent in DM2 without complications. Based on the speculations above, SAA might be a novel and promising drug candidate for diabetes treatment, which may eventually contribute to the amelioration of the heavy burden of CVD among the population of DM2. Moreover, to verify all these speculations, further clinical trial investigations will be needed.

There are several limitations to this study. The low number of enrolled patients resulted in there being fewer than 20 in each of the three groups, with only 11 in the group consisting of DM2 patients without diabetic complications. A further limitation is that platelet reactivity was not measured in patients treated with SAA due to a lack of evidence that SAA is safe to use in a human body test.

## Conclusions

In summary, we examined platelet activation using flow cytometry and platelet aggregation, utilizing transmittance aggregometry to assess the antiplatelet effect of SAA in DM2 patients. We found that SAA can inhibit platelet activation and aggregation in patients with DM2, and the inhibition did not abate diabetic complications. SAA thus may be developed as a novel antiplatelet agent, providing an alternative to antiplatelet therapies for DM2.

## Data Availability

Datasets are available from the corresponding author upon reasonable request.

## Abbreviations

ADP: Adenosine diphosphate
AIDS: Acquired immune deficiency syndrome
CD62p: P-selectin
COX-1: Cycloxigenase-1
CVD: Cardiovascular disease
DM2: Type 2 diabetes mellitus
DN: Diabetic nephropathy
DPN: Diabetic peripheral neuropathy
DRP: Diabetic retinopathy
GPVI: Platelet-specific collagen receptor glycoprotein VI
HbA1c: Hemoglobin A1c
HDL: High-density lipoprotein
HIV: Human immunodeficiency virus
LDL: Low-density lipoprotein
MPV: Mean platelet volume
PAC-1: Fibrinogen binding
PDW: Platelet distribution width
PI3K: Phosphoinositide 3-kinase
PLT: Platelet
SAA: Salvianolic acid a
TC: Total cholesterol
TG: Triglyceride
